# Supplementary material for: Achieving HIV epidemic control through integrated community and facility-based strategies: Lessons learnt from ART-surge implementation in Akwa Ibom, Nigeria
Source: PLoS One. 2022 Dec 21;17(12):e0278946. doi: 10.1371/journal.pone.0278946 (PMC9770335; doi:10.1371/journal.pone.0278946)
Supplement: Attachment — Submitted filename: Response to Reviewers.docx [file pone.0278946.s002.docx]

PONE-D-22-14919

Achieving HIV Epidemic Control through integrated community and facility-based strategies: Lessons Learnt from ART-surge Implementation in Akwa Ibom, Nigeria

PLOS ONE

The Editor,

PLOS ONE

The authors thank you and the external reviewers for the detailed evaluation of our manuscript titled: “Achieving HIV Epidemic Control through integrated community and facility-based strategies: Lessons Learnt from ART-surge Implementation in Akwa Ibom, Nigeria” (PONE-D-22-14919) by Nwaokoro et al. The authors have revised the manuscript in keeping with the reviewer’s comments and recommendations and have provided a point-by-point response below. A marked-up copy of the manuscript that highlights changes made to the original version and an unmarked version of the revised paper without tracked changes have also been uploaded.

We look forward to hearing from you. Thank you.

Yours sincerely

Pius Nwaokoro

**RESPONSE TO THE EDITOR ON JOURNAL REQUIREMENTS:**

1. Please ensure that your manuscript meets PLOS ONE's style requirements, including those for file naming. The PLOS ONE style templates can be found at 
<https://journals.plos.org/plosone/s/file?id=wjVg/PLOSOne_formatting_sample_main_body.pdf> and 
<https://journals.plos.org/plosone/s/file?id=ba62/PLOSOne_formatting_sample_title_authors_affiliations.pdf>

1. *Thank you for this feedback. The manuscript has been formatted to meet the requirements.*

2. Thank you for stating the following financial disclosure:

This publication resulted in part from data collected during the implementation of the PEPFAR-funded SIDHAS project in Nigeria (Cooperative Agreement Number: AID-620-A-11-00002).
  
Please state what role the funders took in the study.  If the funders had no role, please state: The funders had no role in study design, data collection and analysis, decision to publish, or preparation of the manuscript." 
If this statement is not correct you must amend it as needed. 
Please include this amended Role of Funder statement in your cover letter; we will change the online submission form on your behalf.

2. *Thank you for this observation. The financial disclosure segment has been updated to include the statement: ”The funders had no role in study design, data collection and analysis, decision to publish, or preparation of the manuscript."*

3. We note that Figure 1 in your submission contain [map/satellite] images which may be copyrighted. All PLOS content is published under the Creative Commons Attribution License (CC BY 4.0), which means that the manuscript, images, and Supporting Information files will be freely available online, and any third party is permitted to access, download, copy, distribute, and use these materials in any way, even commercially, with proper attribution. For these reasons, we cannot publish previously copyrighted maps or satellite images created using proprietary data, such as Google software (Google Maps, Street View, and Earth). For more information, see our copyright guidelines: <http://journals.plos.org/plosone/s/licenses-and-copyright>.
 
We require you to either (1) present written permission from the copyright holder to publish these figures specifically under the CC BY 4.0 license, or (2) remove the figures from your submission:
 
a. You may seek permission from the original copyright holder of Figure 1 to publish the content specifically under the CC BY 4.0 license.  
 
We recommend that you contact the original copyright holder with the Content Permission Form (<http://journals.plos.org/plosone/s/file?id=7c09/content-permission-form.pdf>) and the following text:
“I request permission for the open-access journal PLOS ONE to publish XXX under the Creative Commons Attribution License (CCAL) CC BY 4.0 (<http://creativecommons.org/licenses/by/4.0/>). Please be aware that this license allows unrestricted use and distribution, even commercially, by third parties. Please reply and provide explicit written permission to publish XXX under a CC BY license and complete the attached form.”
 
Please upload the completed Content Permission Form or other proof of granted permissions as an "Other" file with your submission.
 
In the figure caption of the copyrighted figure, please include the following text: “Reprinted from [ref] under a CC BY license, with permission from [name of publisher], original copyright [original copyright year].”
 
b. If you are unable to obtain permission from the original copyright holder to publish these figures under the CC BY 4.0 license or if the copyright holder’s requirements are incompatible with the CC BY 4.0 license, please either i) remove the figure or ii) supply a replacement figure that complies with the CC BY 4.0 license. Please check copyright information on all replacement figures and update the figure caption with source information. If applicable, please specify in the figure caption text when a figure is similar but not identical to the original image and is therefore for illustrative purposes only.
The following resources for replacing copyrighted map figures may be helpful:
 
USGS National Map Viewer (public domain): <http://viewer.nationalmap.gov/viewer/>
The Gateway to Astronaut Photography of Earth (public domain): <http://eol.jsc.nasa.gov/sseop/clickmap/>
Maps at the CIA (public domain): <https://www.cia.gov/library/publications/the-world-factbook/index.html> and <https://www.cia.gov/library/publications/cia-maps-publications/index.html>
NASA Earth Observatory (public domain): <http://earthobservatory.nasa.gov/>
Landsat: <http://landsat.visibleearth.nasa.gov/>
USGS EROS (Earth Resources Observatory and Science (EROS) Center) (public domain): [http://eros.usgs.gov/#](http://eros.usgs.gov/)
Natural Earth (public domain): <http://www.naturalearthdata.com/>

3. *Thank you for the observation. The authors have removed the image and all reference to it within the text. The authors believe that the removal of the image will not considerably affect the reader’s understanding of the implementation environment. In addition, the image labeled “Fig 2” has now been edited to “Fig 1” and likewise for “Fig 3” to maintain consistency. References to these figures within the text have also been updated accordingly.*

4. Please include captions for your Supporting Information files at the end of your manuscript, and update any in-text citations to match accordingly. Please see our Supporting Information guidelines for more information: <http://journals.plos.org/plosone/s/supporting-information>.

4. *Thank you for this observation. The manuscript do not have support information files.*

**RESPONSE TO REVIEWERS**

**Reviewer #1:**

1. Lines 34 - 36: It was stated that ANC sentinel survey and NAHRS over estimated prevalence and number of PLHIV. The statemen is true for prevalence but not completely accurate for number of PLHIV because these 2 surveys do not directly estimate PLHIV population. PLHIV population is estimated using a model. Please correct the statement.

*We thank the reviewer for this helpful comment. We have now addressed this by removing the statement on number of PLHIV (3.2million).*

2. Line 40: The same comment above applies to NAIIS survey.

*We appreciate the reviewer for this helpful comment. We have referenced the 2019 spectrum data which provides the PLHIV estimate for Nigeria.*

3. The statement “AkwaIbom ranks highest in HIV prevalence in Nigeria” is a very strong statement and is one of the major findings of NAIIS. I wonder why the authors decided to use online newspaper as reference instead of the primary reference (NAIIS) which is more scientific. Consider using NAIIS report please.

*The authors agree that NAIIS is a stronger reference and have made the adjustment on the manuscript*

4. Line 67: It will be good to describe the peculiarities of the two phases of the Surge Apr 2019 – Sep 2019 vs Oct 2019 – Mar 2021. Why is it in two phases, why the scale down in No. of LGAs in the 2nd phase? Additionally, the 102 facilities involved are they from both phases or from the 2nd phase only?

*We have revised the manuscript to clarify the difference between the two phases. In phase 1 the project supported a larger catchment area. From October 2019, PEPFAR the funding agency introduced a new partner to cater for 10 LGAs which were removed from the catchment area previously supported by the project. The 102 facilities were involved in both phases*

5. Line 79: use of risk stratification tool; please qualify as use of HIV risk stratification tool. Additionally, it will be good to define the tool and its purpose or reference where the reader can get details about the tool.

*The authors have added references that describes the tool and its use in improving HIV testing efficiency.*

6. Line 83: results were promptly returned to providers through an online platform. This is very important point and I recommend the authors to explain the online platform and its functionality. The objective of this paper is to provide lessons learnt. Therefore, the authors should please not assume that the readers are familiar with everything stated.

*Thank you for this insightful feedback, we have expanded our narrative to include the use of the online laboratory information management system used to return viral load results from the reference Laboratory to the service providers.*

7. Line 87: Surge framework – this is an important component in the surge and should have been introduced early. For example, this could have been at the beginning of the subsection – Surge Intervention. Something like “the Surge intervention was based on EPIC framework ……………., then sentence in line 78 can follow. Additionally, the Surge Framework should be introduced in the abstract.

*We agree with the reviewer, and we have adjusted the manuscript to align with the recommendation. See line 77 in the revised track changes version of the manuscript.*

8. Line 92: the sentence “focus group discussions with clients to identify existing barriers to implementation in their...” is hanging, incomplete.

*We thank the reviewer for highlighting this, we have revised the sentence and it reads as follows “focus group discussions were held with clients to identify existing barriers to implementation, in their as well as brainstorming sessions…”*

9. Figure 2 caption should be more elaborate.

*We agree with the reviewer’s comment and have revised the caption of this figure. However, in the revised manuscript it is Figure 1 and reads “Adapting EPIS framework for the ART surge implementation in Akwa Ibom, Nigeria (April 2019 – March 2021)*AKAIS: Akwa Ibom AIDS Indicator Survey”*

10. Lines 101 – 105: It will be good to understand how many LGAs per cluster, how many CAM and sub teams. This level of granularity is important for the reader to understand the complexity of the operations deployed for the Surge. Use of “several” may not be adequate

*We thank the reviewer for this insightful comment, we have indicated the number of LGA, and CAM teams to give the reader a better understanding of the context under which the surge was implemented.*

11. Line 97: The title of Table 1 is a bit misleading when compared to the statement where table 1 was referenced. The statement says “The merits and demerits of each EBP were considered based on implementation experience and adapted following established approaches [12]. Table 1 shows the EBPs that were implemented”. Is the EPIC synonymous with the EBP? Consider revising the title and if possible, remove EPIC or introduce EPIC in line 97 to ensure consistency with the table title.

*Thank you for this insightful feedback. The authors have revised the table title and removed the reference to “ERIC” to provide the needed clarity.*

12. Table 1 is too long and could be more concise. Some EBPs could be merged into a single EBPs. Similarly, SIDHAS strategies could also be regrouped based on strategies that are similar thereby shortening the table. Due to length of the table and that the strategies are not systematically presented, this push the reader to be flipping up and down trying to align strategies that are similar in order to contextualize the approach.

*The authors appreciate this feedback. The table has been revised and made more concise.*

13. Line 124: “those with the highest client volume received volume-intensive support”. Phrase not clear.

*The authors appreciate the recommendation, and have rephrased the statement indicate the maximal support to these sites called the enhanced site management (ESM) facilities*

14. Line 131 and table 2: Reaching 1st 95% - very important points have been raised. It will be appropriate if the authors will provide additional context on how several strategies here were achieved or they should provide references so that readers can get in-depth understanding on how these strategies can be replicated. For example, HIV self-testing, geo-targeting of hotspots, engaging private labs, patent medical vendors TBAs e.t.c.

*The authors appreciate this feedback and have made reference to the project technical implementation guide (reference 17) . We hope that this reference addresses the reviewer’s comment.*

15. Line 155: “clients received multi month dispensing (MMD) with refill frequencies of 3, 3-5 and 6-monthly in any of the models”. Will be good to describe under what circumstances the the decision to use 3-, 3-5- and 6-month MMDs. Alternatively provide reference.

*The authors appreciate this feedback and have made some revisions to the manuscript and reference to aa publication by the same authors which describes multimonth dispensing and other client centered differentiated service delivery models.*

16. Line 182: Data collection: Since DHIS, DATIM are National and PEPFAR data management platforms, the authors may wish to consider making this clearer by stating that SIDHAS leveraged National and PEPFAR data management platforms, DHIS and DATIM respectively to …….

*Thank you for this insightful feedback. The authors have adopted this recommendation and rephrased the subject line as suggested by the reviewer.*

17. Please correct definition of DATIM from “Data for Accountability, Integrity, and Management” to “Data for Accountability, Transparency and Impact”. Also provide reference.

*Thank you for highlighting this error. We apologize for the misrepresentation and have made*

18. Line 182. Data collection. It will be good for the authors to be very explicit in this section by listing the variables and indicators that were collected. Additionally, DHIS and DATIM were mentioned in the 1st paragraph, but DATIM was never mentioned again. What data goes in to DATIM and what role did DATIM played in this data collection. Additionally, LAMIS was mentioned but then EMR was mentioned later. Please be consistent on whether to use LAMIS or EMR as not all readers may understand both mean the same.

*Thank you for this observation. The variables and indicators have been listed. Validated data from DHIS and LAMIS is reported to the donors in DATIM quarterly and this data is used for routine monitoring of program implementation. The reference to EMR has been removed from the manuscript with LAMIS used all throughout for consistency.*

19. Line 197: Please define “key outcomes” by listing them

*Thank you for this observation. The outcomes have been listed and appear under lines 214-217 of the revised manuscript.*

20. Line 203: This statement is a bit confusing “Clients were considered to be retained in care if their next ART pickup date was after March 31, 2019, for the pre-surge cohort and March 31, 2021, for the surge cohort”. Please provide definition of retention in the context of this study in number of months and also provide starting period for both pre-surge and surge cohort periods.

*Thank you for this observation. Retention was determined at 12 months after ART commencement. Clients who missed a clinic appointment and did not return 28 days after expected clinic were considered “not retained” at 12 months. The manuscript has been revised to reflect this.*

21. Line 210: Data for this study were collected from an existing project database used for routine program monitoring. Is this database different from DHIS and DATIM earlier stated in data analysis section? If yes, then it may be okay to remove DATIM and DHIS from the data analysis section and to use project database.

*Thank you for this observation. The database referenced here is the same as DHIS and DATIM. The manuscript has been updated by removing the reference to a database*

22. Results, Table 3: In line 102, Oron, Eket, and Uyo were listed as the only 3 clusters for the Surge. Here you added Ikot Ekpene cluster as additional cluster in pre-Surge. To enable good comparison (pre-Surge and Surge periods) and to minimize confusions, please consider dropping the Ikot Ekpene from Table 3.

*Thank you for this suggestion. The authors have redone the analysis, revised table 3 and updated the manuscript to reflect this.*

23. Results: Table 3 contradicts Table 2 (testing modality and testing strategy). In Table 2, for example community testing was not part of pre-Surge activities but in Table 3 results were provided under community testing. Also, in Table 2, Genealogy and Community Hotspot testing were indicated but no results indicated in Table 3. Please reconcile. If no data for these strat. egies, then include this under limitations.

*The authors appreciate the reviewer for this observation. We have revised the table 3 to include data for Community Hotspot testing and Table 2 to include other strategies whose results have been reported in table 3. The reference to genealogy in the manuscript was as a component of index testing and data for index testing is presented in Table 3. Also, the inclusion of community testing data as part of the pre-surge activities was done in error and has been removed from the table.*

24. Results, Table 3: Positivity rates are inconsistently presented to either whole number or 2 decimal percentages. Please present all positivity rates to 1 decimal percentage. This is how prevalence and testing yields are standardly presented.

*Thank you for this observation. The manuscript has been updated to reflect this.*

25. Discussions: Line 298 “This endeavor helped increase the treatment coverage across 21 high burden local government areas and the overall treatment coverage to 87.8%”. These findings were not presented in results section and may not be discussed under the discussions section. If the authors decided to discuss this, then they should indicate LGA level coverage in the results. Additionally, baseline coverage should be presented so that increase in coverage over the 24 months (Surge period) can be appreciated.

*Thank you for this observation. The authors agree that the LGA coverage was not reported in the results segment and have therefore removed any reference to it in the discussion.*

26. Discussions: Line 309 “…….and introduced an innovative Community ART Management (CAM) approach for timely testing, linkage, and to provide support to clients on ART”. The sentence seems grammatically in correct, after “for” “to provide” was used. Consider “…….and introduced an innovative Community ART Management (CAM) approach for timely testing, linkage, and support to clients on ART”.

*Thank you for this feedback. The manuscript has been updated to reflect his correction.*

27. Discussions: Line 322 “We improved the impact of this strategy by targeting people who were on treatment but virally unsuppressed”. To aid clarity add “…. the partners of people…” because it is the partners of unsuppressed individuals on ART that are targeted.

*Thank you for this observation. the manuscript has been updated to provide this clarification.*

28. Additionally, does the result indicate how adding partners of unsuppressed individuals on ART affect the index testing strategy? What is the contribution of partners of unsuppressed individuals to the total positives identified through index testing?

*Thank you for this feedback. Our analysis did not disaggregate the contribution of the partners of unsuppressed individuals to the total positives identified through index. This has been included as a limitation of the study.*

29. Discussions: Line 326 “Effective targeting or geographies with high-risk individuals and timing or testing times helped achieve a high positivity rate of 5.5% at the community level”. Your result (Table 3) shows 5.3% and not 5.5%. Additionally, pre-Surge community testing yield was just 1.0%. I wonder why the authors didn’t mention this comparison here. Although, in my earlier comment (No.23) above, I requested the authors to reconcile Tables 2 and 3 whereby strategies not mentioned in the methods were shown in results and community testing during pre-Surge was one of them.

*Thank you for this observation. The positivity rate is actually 5.5% and has been corrected in the manuscript. Table 3 has been updated based on your earlier feedback and the pre-surge community component has been removed.*

30. Discussions: Line 328 “We maintained a high positivity yield throughout the surge period through a mixture of strategies, reviewing data, and use of HIV risk screening before HIV testing which ensured that only high–risk persons in the community setting were tested”. The authors may wish to reference a similar study in Nigeria Surge which used the same strategies. This will diversify reader’s understanding of this paper and the strategies described. See: doi: 10.2147/HIV.S316480. eCollection 2021.

*Thank you for this observation. The authors have added the suggested reference.*

**Reviewer #2:**

Nwaokoro and colleagues summarize the impressive programmatic results of a "surge" initiative in Nigeria. Compared to pre-surge, HIV clinical cascade, there were significant and substantial increases in case identification, linkage to ART, same-day ART, retention and viral load suppression -- all in the face of COVID-19. It was also notable that the large increase in cases identified was not simply the result of indiscriminate massive-volume HIV testing, but rather strategic testing mix which resulted in an increase in test positivity even as testing volumes increased. The paper is well organized and clearly written overall.

Major comments:

(1) It would be helpful if the authors included information about the funding levels during the pre-surge and surge periods. How the did the relative increase in funding during surge compare to the relative increases in cases identified and patients with VL suppression?

*Thank you for this observation. At the time of writing, the authors do not have access to this information to direct costs and have included this as part of the recommendations.*

(2) Did the program scale up TLD and other DTG-based regimens during the surge? This would be a potentially major contributor to the increase in VL suppression during the surge that may should be accounted for in the results and discussion.

*Thank you for the observation. The authors agree that the use of DTG-based regimen could have been a major contributor to the increased VL suppression and have updated the manuscript to reflect this*

(3) There was a large number of interventions and activities implemented as part of the surge. It would be helpful to know if the authors were able to determine which interventions/activities appeared to account for relatively greater impact on clinical outcomes. This may be something that should be added to the limitations.

*Thank you for this observation. the authors do not have sufficient data to make this determination and have updated the manuscript to include it as part of the limitations of the study.*

Minor comments:

(1) p 11. The authors indicate that EAC was provided if the VL was >1000. In that situation, how soon was the VL repeated?

*Thank you for this observation. VL was repeated after 3 months of EAC. The manuscript has been updated to reflect this.*

(2) Table 3. The % POS during surge should be 5%, not 4%. In the text of the results (p 13), it would be good to mention that % POS increased even with dramatic increases in testing volumes, an important achievement.

*Thank you for the observation. The authors have updated this section of the manuscript to reflect this.*

(3) It is even more remarkable that these results were achieved while COVID-19 was raging. Consider adding that context more clearly to the discussion.

*Thank you for the observation. The authors have updated this section of the manuscript to reflect this.*
